# 44190 Comparing the Accuracy of Different Tools in Identifying Glaucoma Medication Non-adherence

**DOI:** 10.1017/cts.2021.725

**Published:** 2021-03-30

**Authors:** Juno Cho, Leslie Niziol, Paul Lee, Michele Heisler, Kenneth Resnicow, David C Musch, Paula Anne Newman-Casey

**Affiliations:** 1 Michigan Medicine; 2 University of Michigan School of Public Health

## Abstract

ABSTRACT IMPACT: Medication non-adherence is a widespread problem in glaucoma care, and this abstract shows that a free and easy to implement tool can be used to accurately screen and identify patients who are not adherent to their glaucoma medication. OBJECTIVES/GOALS: To compare the accuracy of pharmacy refill data and five measures of self-reported adherence in identifying patients with poor electronically monitored glaucoma medication adherence. METHODS/STUDY POPULATION: Glaucoma patients (age ≥40, poor self-reported adherence, and ≥1 medication) recruited at the University of Michigan completed five surveys of adherence and 3-months of electronically monitored medication adherence; pharmacy refill data were obtained. Electronically monitored adherence was summarized monthly as percent of doses taken on time. Median monthly adherence ≤80% was considered non-adherent. Pharmacy refill data were reported as the proportion of days covered. The accuracy of the measures in predicting ≤80% adherence was assessed with receiver operating characteristic curves such as estimation of area under the curve (AUC), sensitivity, specificity, and accuracy. RESULTS/ANTICIPATED RESULTS: 95 patients completed electronic monitoring with a median monthly adherence of 74% (±21%); 53 patients (56%) were non-adherent. Pharmacy refill adherence was not significantly correlated with electronically monitored medication adherence (r=0.12, p=0.2). A single-item adherence question (‘Over the past month, what percentage of your drops do you think you took correctly?’) had the largest correlation with electronically monitored adherence (r=0.47, p<0.0001), the largest AUC for predicting non-adherence (AUC= 0.76, [95% Confidence Interval = 0.66, 0.87]), best accuracy (71%, [61, 82]), and good sensitivity (84%, [73, 96]). DISCUSSION/SIGNIFICANCE OF FINDINGS: A free, single-item screening question ('Over the past month, what percentage of your drops do you think you took correctly?') offers an easy-to-implement tool for identifying glaucoma patients with poor medication adherence in clinical practice.

